# Editorial: Encapsulated probiotics: next-generation functional food production

**DOI:** 10.3389/fmicb.2026.1915068

**Published:** 2026-07-09

**Authors:** Kandi Sridhar, Shweta Suri, Minaxi Sharma

**Affiliations:** 1 Department of Food Technology, Karpagam Academy of Higher Education (Deemed to be University), Coimbatore, India; 2 School of Health Sciences and Technology, University of Petroleum and Energy Studies (UPES), Dehradun, India; 3 Center for Life Science and Healthcare, Nottingham Ningbo China Beacons of Excellence Research and Innovation (CBI), University of Nottingham Ningbo China, Ningbo, China

**Keywords:** encapsulated probiotics, functional foods, microencapsulation, probiotic delivery systems, probiotic stability

The increasing global demand for healthier foods and preventive healthcare strategies has accelerated the development of functional foods enriched with bioactive ingredients. Among these, probiotics have gained considerable scientific and industrial attention due to their demonstrated benefits in maintaining gut health, modulating immune responses, improving metabolic functions, and contributing to overall wellbeing. Beyond their traditional applications in digestive health, emerging evidence suggests that probiotics may play significant roles in weight management, cardiovascular health, mental wellness, and the prevention of various chronic diseases. Consequently, the incorporation of probiotics into food systems has become a major focus for food researchers/scientists, microbiologists, and the functional food industry.

Despite their recognized health-promoting properties, the successful application of probiotics in food products remains challenging. Probiotic microorganisms are highly sensitive to environmental stresses encountered during food processing, storage, transportation, and gastrointestinal transit. For example, temperature fluctuations, oxygen exposure, moisture, acidity, and digestive enzymes can significantly reduce probiotic viability, thereby limiting their functionality and health benefits. Therefore, ensuring the stability, survival, and targeted delivery of probiotic cells has become a critical prerequisite for the development of next-generation functional foods.

To this end, encapsulation technologies have emerged as one of the most promising strategies to overcome these challenges. By entrapping probiotic cells within protective matrices, encapsulation techniques enhance cell survival during processing and storage while facilitating controlled release at specific sites within the gastrointestinal tract. Various encapsulation approaches, including extrusion, emulsion, spray drying, spray chilling, fluidized-bed coating, coacervation, and nanoencapsulation have demonstrated significant potential in preserving probiotic viability and functionality. In particular, advances in micro/nano encapsulation technologies have enabled the development of sophisticated delivery systems capable of improving probiotic bioavailability, stability, and therapeutic efficacy. Furthermore, the integration of encapsulated probiotics into diverse food matrices offers broader opportunities for designing innovative functional foods with enhanced nutritional and health-promoting attributes.

Recent advancements in materials science, food engineering, microbiology, and nanotechnology have further expanded the scope of probiotic encapsulation research. Novel biopolymeric carriers, smart delivery systems, and multifunctional encapsulation matrices are being investigated to improve probiotic protection and targeted release. Additionally, encapsulated probiotics are increasingly being explored as natural antioxidants, antimicrobial agents, and active components in intelligent food packaging systems. Nevertheless, several scientific and technological challenges remain, including large-scale production, cost-effectiveness, regulatory considerations, consumer acceptance, and maintaining probiotic functionality throughout the product lifecycle. Addressing these challenges requires interdisciplinary collaboration among researchers, food manufacturers, and healthcare professionals. Thus, to advance scientific understanding and foster innovation in this rapidly evolving field, a team of scientists (Kandi Sridhar, Ph.D., Shweta Suri, Ph.D., and Minaxi Sharma, Ph.D.) with expertise in functional foods, food microbiology, probiotic delivery systems, and encapsulation technologies, organized the riveting Research Topic collection entitled “*Encapsulated probiotics: next-generation functional food production*” to be published in *Frontiers in Microbiology* (ISSN 1664-302X), a leading international journal dedicated to advancing microbial sciences. This Research Topic aims to provide a comprehensive platform for disseminating cutting-edge research and technological developments related to encapsulated probiotics and their applications in functional food production.

The Research Topic encompasses a wide range of contemporary themes, including but not limited to:

- Novel probiotics and their applications in functional food production.
- Micro/nano nanoencapsulation technologies for probiotic delivery.
- Influence of encapsulation strategies on probiotic stability and functionality.
- Bioactivity, bioavailability, and controlled delivery of probiotics in diverse food matrices.
- Encapsulated probiotics as natural antioxidants and antimicrobial agents in foods.
- Probiotic-based smart and active packaging systems.
- Emerging challenges, opportunities, and future prospects of probiotic incorporation into innovative functional foods.

This Research Topic has attracted contributions from researchers worldwide, reflecting the growing importance of encapsulated probiotics in food innovation and human health. The published articles collectively provide valuable insights into novel encapsulation approaches, advanced delivery systems, probiotic functionality, and industrial applications. After rigorous peer review, four articles were accepted and published in this Research Topic. One of the studies under this Research Topic, Liu et al. investigated the molecular mechanisms underlying stress resistance in the probiotic strain *Limosilactobacillus reuteri* FP41 by focusing on its ability to withstand acid, bile salt, and freeze-drying stresses encountered during food processing and gastrointestinal transit. Through transcriptomic and proteomic analyses, the authors identified three key stress-response genes (csbD, osmC, and gatD) that were significantly upregulated under adverse conditions. Functional validation demonstrated that overexpression of these genes markedly improved probiotic survival under simulated gastric conditions, bile salt exposure, and vacuum freeze-drying. Complementing this work, Cui et al. developed an innovative vacuum freeze-dried bacterial tablet platform designed to improve bacterial preservation and facilitate culture medium evaluation. Using response surface methodology, the authors optimized a composite cryoprotectant system comprising glycerol, brain heart infusion, sucrose, and sodium glutamate, achieving bacterial survival rates exceeding 90% post-lyophilization. The study further demonstrated that storage under vacuum at −20 °C effectively maintained bacterial viability over extended periods. Importantly, the freeze-dried bacterial tablet method exhibited high sensitivity and specificity when compared with the ISO 11133-2014 standard for culture medium evaluation. Together, these studies address two critical challenges in probiotic commercialization (i.e., cellular stress resistance and preservation stability) and contribute valuable knowledge toward the development of efficient probiotic delivery systems and next-generation functional foods.

Cardoso-Cardenas et al. and Huang et al. collectively highlight the critical role of both advanced encapsulation technologies and next-generation probiotic strains in the development of functional foods. Cardoso-Cardenas et al. demonstrated that optimized microencapsulation strategies effectively preserved the viability and functionality of *Limosilactobacillus fermentum* K73 during processing and simulated gastrointestinal digestion, maintaining key probiotic attributes such as bile salt hydrolase activity, cholesterol assimilation, epithelial adhesion, and hypocholesterolemic efficacy. Complementing these findings, Huang et al. characterized AKM Lab-01, a novel *Akkermansia muciniphila* strain with potent anti-obesity and metabolic health benefits, showing that both live and processed forms retained their probiotic functionality and safety while improving body weight, glucose metabolism, and lipid profiles in diet-induced obese mice. Together, these studies underscore the importance of integrating robust probiotic delivery systems with scientifically validated probiotic strains to enhance stability, bioactivity, and health outcomes, thereby advancing the production of next-generation functional foods targeting metabolic and cardiovascular health.

In conclusion, the studies published in this Research Topic provide important advances toward the development of next-generation probiotic-based functional foods. The contributions collectively address key challenges associated with probiotic commercialization, including stress tolerance, viability, preservation, and delivery. The findings presented in this Research Topic contribute valuable knowledge toward improving probiotic stability, viability, and industrial applicability, thereby supporting the development of innovative, sustainable, and consumer-oriented functional food products.

As Guest Editors, we are delighted to present this Research Topic of articles to the scientific community through Frontiers in Microbiology. We believe that this Research Topic provides a valuable platform for researchers, industry stakeholders, and healthcare professionals interested in probiotic technologies and functional food innovation. Furthermore, we anticipate that the findings reported herein will stimulate future investigations into advanced encapsulation strategies, smart delivery systems, strain engineering approaches, and novel probiotic applications. We hope that this Research Topic will serve as a foundation for continued research and technological developments that accelerate the translation of probiotic science into effective next-generation functional food products with enhanced health benefits and commercial potential.

